# Active Hydrophilic Components of the Medicinal Herb *Salvia miltiorrhiza* (Danshen) Potently Inhibit Organic Anion Transporters 1 (Slc22a6) and 3 (Slc22a8)

**DOI:** 10.1155/2012/872458

**Published:** 2012-07-15

**Authors:** Li Wang, Douglas H. Sweet

**Affiliations:** Department of Pharmaceutics, Virginia Commonwealth University, 410 N 12th Street, Richmond, VA 23298, USA

## Abstract

Many active components of herbal products are small organic anions, and organic anion transporters were previously demonstrated to be a potential site of drug-drug interactions. In this study, we assessed the inhibitory effects of six hydrophilic components of the herbal medicine Danshen, lithospermic acid, protocatechuic acid, rosmarinic acid, salvianolic acid A, salvianolic acid B, and tanshinol, on the function of the murine organic anion transporters, mOat1 and mOat3. All of Danshen components significantly inhibited mOat1- and mOat3-mediated substrate uptake (*P* < 0.001) with lithospermic acid (LSA), protocatechuic acid, rosmarinic acid (RMA), and salvianolic acid A (SAA) producing virtually complete inhibition under test conditions. Kinetic analysis demonstrated that LSA, RMA, and SAA were competitive inhibitors. As such, *K*_*i*_ values were estimated as 14.9 ± 4.9 **μ**M for LSA, 5.5 ± 2.2 **μ**M for RMA, and 4.9 ± 2.2 **μ**M for SAA on mOat1-mediated transport, and as 31.1 ± 7.0 **μ**M for LSA, 4.3 ± 0.2 **μ**M for RMA, and 21.3 ± 7.7 **μ**M for SAA on mOat3-mediated transport. These data suggest that herb-drug interactions may occur *in vivo* on the human orthologs of these transporters in situations of polypharmacy involving Danshen and clinical therapeutics known to be organic anion transporter substrates.

## 1. Introduction

The Chinese herbal medicine, Danshen (*Salvia miltiorrhiza*), has been employed for thousands of years in the relief of symptoms of cardiovascular disease [1–3]. Despite this long history of medicinal use, the issue of which component(s) is (are) responsible for its therapeutic effects, and the precise biochemical mechanisms underlying their absorption, distribution, and elimination, remain largely unknown. Increasingly, six hydrophilic compounds, lithospermic acid (LSA), protocatechuic acid (PCA) (in this work we use the abbreviation PCA to designate protocatechuic acid. Some previous reports have used PCA to refer to protocatechuic aldehyde. Protocatechuic aldehyde is a major component in Danshen extracts whereas the protocatechuic acid level is insignificant), rosmarinic acid (RMA), salvianolic acid A (SAA), salvianolic acid B (SAB), and tanshinol (TSL), are gaining favor as the Danshen components responsible for the beneficial effects on heart disease [4–6]. Since these compounds are organic, small in size (154–718 Da), and exist as anions at physiological pH, it is possible that they are substrates and/or inhibitors of the organic anion transport pathway that exists in organs such as the kidney, liver, intestine, and choroid plexus [7].

We now know that this organic anion transport pathway is actually a complex system of transport proteins that belong to a variety of gene families. Members of the solute carrier (SLC) superfamily, composed of ~55 gene families containing almost 400 identified transporters, are an important component of this pathway [7–9]. Of particular interest for the Danshen components examined in this work is the SLC22 (organic cation/anion/zwitterion transporters) family, which includes both the organic cation transporters and the organic anion transporters (OATs). Members of the OAT family are found in virtually every barrier tissue in the body and mediate the transepithelial flux (absorption, distribution, and elimination) of a multitude of endogenous and xenobiotic compounds [7–9]. Typical endogenous OAT substrates include sulfated steroid conjugates, indoxyl sulfate, uric acid, and acidic metabolites of monoamine neurotransmitters. Xenobiotic substrates include clinically important therapeutics such as antibiotic (benzylpenicillin), antiviral (adefovir, cidofovir) and anticancer (methotrexate) agents, statins, and angiotensin-converting enzyme inhibitors, as well as environmental toxins such as ochratoxin A and aristolochic acid [7, 9, 10].

Currently, there are 29 putative SLC22 family members, 18 of which are believed to be OATs. Of those, transport activity has been demonstrated for eleven, OAT1-10 and URAT1 [7, 9]. OATs expressed in the proximal tubule cells of the kidney mediate the blood to urine secretory flux and urine to blood reabsorptive flux of substrate compounds [7–9]. Since these compounds are negatively charged, OATs provide a passageway through which they can readily cross the lipid bilayer of cells. Substrate entry into proximal tubule cells from the blood across the basolateral membrane is energized by exchange for intracellular *α*-ketoglutarate (i.e., organic anion/dicarboxylate exchange). In humans and rodents this process involves the basolateral exchangers and organic anion transporter 1 (Oat1; Slc22a6) and 3 (Oat3; Slc22a8) [11, 12]. OAT-mediated exit of organic anions from the cell into the urine is accomplished via facilitated diffusion using the electrochemical gradient. There appears to be distinct species differences between rodents and humans in terms of OATs targeted to the apical membrane of proximal tubule cells. For example, while both species express Urat1 (Slc22a12), humans also have OAT4 (SLC22A9; no current rodent ortholog), whereas rodents have Oat5 (Slc22a19; no human ortholog identified) [7, 9].

The basolateral uptake transporters Oat1 and Oat3 share a high degree of amino acid sequence identity (mOat1 versus mOat3 = 48% and hOAT1 versus hOAT3 = 50%). This protein homology likely contributes to the greatly overlapping substrate profiles exhibited by these two transporters. However, despite this general similarity, the individual affinities for the same substrate often differ greatly between Oat1 and Oat3 [7]. Further, unique substrates that interact with Oat1, but not with Oat3 (and vice-versa) have been identified [7]. Preclinical *in vivo* studies utilizing knockout mouse lines have also demonstrated that, in terms of renal transport function, expression of Oat1 does not fully compensate for loss of Oat3 (and vice-versa) [13–16]. For example, Oat1 knockout mice exhibited complete loss of active tubular *para*-aminohippuric acid (PAH) secretion and decreased renal clearance of the diuretic, furosemide, which was accompanied by significantly decreased efficacy (monitored as decreased natriuresis and increased ED_50_) [16]. Pharmacokinetic studies performed in Oat3 knockout mice detected reduced clearance and increased plasma half-life of benzylpenicillin, ciprofloxacin, and methotrexate [13–15]. However, PAH clearance was unaffected in these animals [14].

To more fully understand the therapeutic efficacy (and/or adverse effects) of herbal products and optimize their clinical use, a more thorough identification of the active components they contain and greater knowledge of the mechanisms determining their pharmacokinetic and pharmacodynamic properties, for example, transporter interactions, need to be elucidated. The aim of the present study was to characterize the effects of six active hydrophilic Danshen components on the transport activity of mOat1 and mOat3. Danshen components producing the greatest inhibition were examined further in studies designed to elucidate the mechanism of inhibition (competitive versus noncompetitive versus uncompetitive). Combining this information with dose response data, we derived inhibitory constants (*K*_*i*_ values). Evidence was gathered showing that LSA, RMA, and SAA serve as potent competitive inhibitors of mOat1 and mOat3 and indicating the potential for marked herb-drug interactions, such as altered pharmacokinetics and pharmacodynamics of coadministered clinical therapeutics that are OAT substrates.

## 2. Materials and Methods

### 2.1. Purified Chemicals

**Figure 1:**
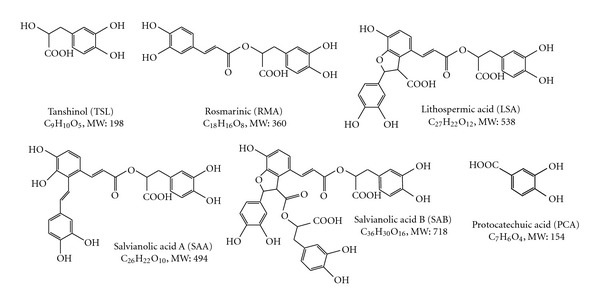
Chemical structures of six active hydrophilic Danshen components. MW: molecular weight.

The Danshen components LSA, PCA, RMA, SAA, SAB, and TSL (≥96% purity) were obtained from Tauto Biotech (Shanghai, China). Their chemical structures are illustrated in Figure 1. Tritiated PAH ([^3^H]PAH) and estrone sulfate ([^3^H]ES) were purchased from PerkinElmer Life and Analytical Sciences (Waltham, MA) and unlabeled PAH, ES, and probenecid were purchased from Sigma-Aldrich (St. Louis, MO, USA).

### 2.2. Tissue Culture

Derivation of the stably transfected Chinese hamster ovary (CHO) cell lines expressing mOat1 (CHO-mOat1), mOat3 (CHO-mOat3), and the empty vector (FRT) transfected control cell line (CHO-FRT) was described previously [13, 15]. Cell lines were maintained at 37°C with 5% CO_2_ in DMEM F-12 media (Mediatech, Inc., Herndon, VA, USA) containing 10% serum, 1% Pen/Strep, and 125 *μ*g/mL hygromycin B.

### 2.3. Cell Accumulation Assays

Cell transport assay procedures were adapted from those previously published [15, 17]. In brief, 2 × 10^5^ cells/well were seeded in 24-well tissue culture plates and grown in the absence of antibiotics for 48 hr. On the day of the experiment, cells were equilibrated with transport buffer for 10 min (500 *μ*L of Hanks' balanced salt solution containing 10 mM HEPES, pH 7.4). Equilibration buffer was replaced with 500 *μ*L of fresh transport buffer containing 5 *μ*M [^3^H]PAH or 1 *μ*M [^3^H]ES (0.25 *μ*Ci/mL) with or without inhibitors. After incubation, cells were immediately rinsed three times with ice-cold transport buffer, lysed, and analyzed via liquid scintillation counting. Uptake values were normalized to corresponding total protein content in cell lysates as determined by the Bradford method. Substrate accumulation is reported as picomoles of substrate per milligram protein. Substrate concentration and accumulation time used for kinetic analysis of mOat3 (1 *μ*M ES for 1 min, *K*_*m*_ = 12.2 ± 4.8 *μ*M) were determined previously [15]. Substrate concentration (Figure 3) and accumulation time (data not shown) for mOat1 kinetic analysis (5 *μ*M PAH for 2 min, *K*_*m*_ = 13.0 ± 3.3 *μ*M) were determined in this study. Kinetic calculations were performed using GraphPad Prism Software version 5.0 (GraphPad Software Inc., San Diego, CA, USA). Michaelis constant (*K*_*m*_) values were calculated by nonlinear regression using the Michaelis-Menten model. Mode of inhibition was identified by using mixed model inhibition analysis [18] as follows:

(1) Vmax⁡  Apparent  =Vmax⁡(1+[I]/(α×Ki)),Km  Apparent=Km1+[I]/Ki(1+[I]/(α×Ki)),Y=Vmax⁡  Apparent×xKm  Apparent+x,

where *V*_max⁡_, *K*_*i*_, and *I* represent the maximum transport velocity without inhibitor, the inhibition constant generated from the data set under analysis, and the concentration of inhibitor, respectively. In this study, three curves were constructed (no inhibitor, plus two selected inhibitor concentrations) with uptake of substrate plotted as a function of its concentration for each condition. These untransformed data were fit to the equations shown above using nonlinear regression to estimate the *α* values summarized in Table 1. The parameter, *α*, can then be used to determine the mode of inhibition. When *α* is very large (*α* > 1), it indicates competitive inhibition. Otherwise, it indicates noncompetitive inhibition (*α* = 1) or uncompetitive inhibition (0 < *α* < 1). To estimate *K*_*i*_ values, IC_50_ values were calculated using nonlinear regression and inserted into the Cheng-Prusoff equation: *K*_*i*_ = IC_50_/(1 + [Substrate]/*K*_*m*_) [19]. Results were confirmed by repeating all experiments at least three times with triplicate wells for each data point in every experiment.

### 2.4. Statistics

Data are reported as mean ± S.D. or mean ± S.E.M. as indicated. Statistical differences were assessed using one-way ANOVA followed by post hoc analysis with Dunnett's *t*-test or using Student's unpaired *t*-test, as indicated (*α* = 0.05).

## 3. Results

### 3.1. Inhibition of mOat1 and mOat3 by Hydrophilic Danshen Components

**Figure 2:**
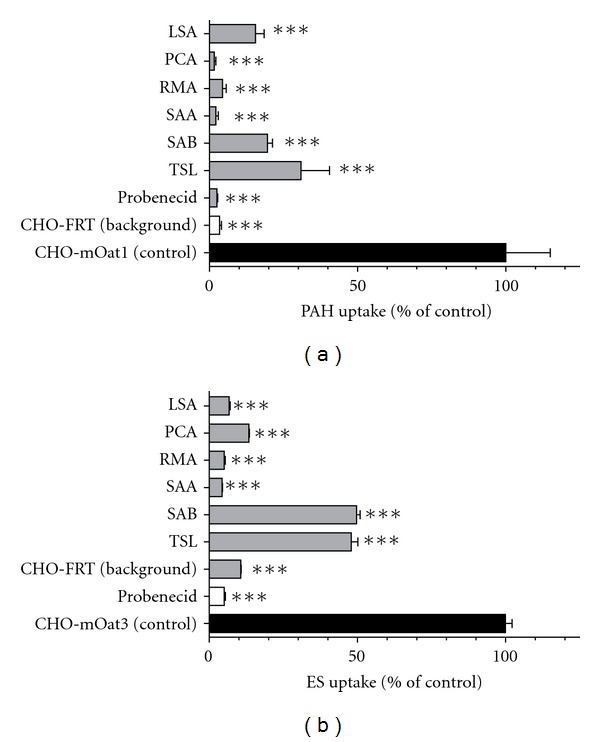
Inhibition profile of mOat1 and mOat3. (a) Inhibition of mOat1-mediated uptake of [^3^H]PAH (5 *μ*M) by LSA, PCA, RMA, SAA, SAB, TSL, and probenecid (1000 *μ*M) was measured in the CHO-mOat1 cells (10 min). Background PAH accumulation was measured in CHO-FRT cells in the absence of inhibitor and is shown to provide a clear gauge of the low background in the experimental system. (b) Inhibition of mOat3-mediated uptake of [^3^H]ES (1 *μ*M) by LSA, PCA, RMA, SAA, SAB, TSL, and probenecid (1000 *μ*M) was measured in the CHO-mOat3 cells (10 min). Background ES accumulation was measured in CHO-FRT cells in the absence of inhibitor and is shown to provide a clear gauge of the low background in the experimental system. Values are mean ± S.D. of triplicate values. ***denotes *P* < 0.001 as determined by one-way ANOVA followed by Dunnett's *t*-test.

Accumulation of PAH in the CHO-mOat1 cell line (98.5 ± 14.6 pmol/mg protein/10 min) was ~30 fold greater than that in the background control CHO-FRT cells (3.3 ± 0.7 pmol/mg protein/10 min; Figure 2(a)). Initially, an uptake assay with excess (1 mM) Danshen components was performed to identify which, if any, of the compounds might interact with mOat1 (Figure 2(a)). Each of the Danshen components, LSA, PCA, RMA, SAA, SAB, and TSL, significantly inhibited PAH uptake in CHO-mOat1 cells (*P* < 0.001) under these conditions. LSA, SAB, and TSL produced approximately 70–85% inhibition, whereas PCA, RMA, and SAA, each reduced PAH accumulation to background level (>95% inhibition), similar to the prototypical OAT inhibitor, probenecid. Further, the addition of these compounds (1 mM) did not significantly influence the low, probenecid-insensitive (i.e., nonspecific) PAH uptake in the CHO-FRT cells (data not shown), indicating that the reduction in uptake of PAH in the CHO-mOat1 cells is attributable to the inhibition of mOat1 activity and that CHO-FRT PAH level serves as an appropriate background correction factor.

Stably transfected mOat3-expressing (CHO-mOat3) cells showed significantly greater accumulation of ES (~20 fold) relative to control CHO-FRT cells (5.02 ± 0.19 versus 0.25 ± 0.04 pmol/mg protein/10 min, resp., Figure 2(b)). Similar to mOat1, all of the Danshen components (1 mM) significantly inhibited mOAT3-mediated ES uptake (*P* < 0.001). SAB and TSL produced approximately 53% and 55% inhibition, respectively. LSA, PCA, RMA, and SAA, like probenecid, blocked virtually all (>91%) mOat3-mediated ES transport (Figure 2(b)). As with PAH, these compounds (1 mM) failed to consistently or significantly influence nonspecific ES uptake in CHO-FRT cells (data not shown), indicating that the reduction in uptake of ES in the CHO-mOat3 cells is attributable to the inhibition of mOat3 activity and that CHO-FRT ES level serves as an appropriate background correction factor.

### 3.2. Determination of the Type of Inhibition Induced by Danshen Components on mOat1 and mOat3

**Figure 3:**
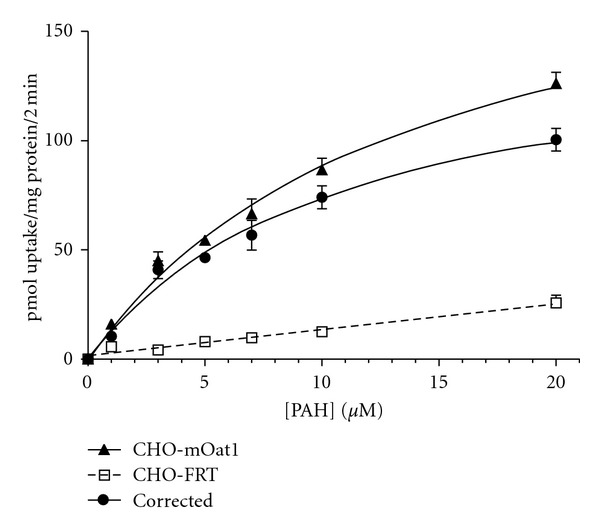
Michaelis-Menten kinetics of PAH transport in CHO-mOat1 cells. In order to calculate *K*_*i*_ values for mOat1, the *K*_*m*_ value for PAH needed to be determined in the CHO-mOat1 cell system. Uptake of [^3^H]PAH was measured for 2 min at room temperature in CHO-mOat1 (closed triangles) and CHO-FRT (open squares) cells in order to construct a saturation curve. The corrected curve (closed circles) was obtained by subtracting the nonspecific background uptake as measured in the CHO-FRT cells from CHO-mOat1 accumulation to allow analysis of mOat1-mediated activity. Experiments were repeated three times in triplicate, and Michaelis constant (*K*_*m*_) values were calculated by nonlinear regression using the Michaelis-Menten model. The *K*_*m*_ for PAH on mOat1 was estimated as 13.0 ± 3.3 *μ*M (mean ± S.E.M.). Graph shown is from a representative experiment with values plotted as mean ± S.D. (*n* = 3).

Although PCA exhibited as potent inhibition as LSA, RMA, and SAA, it is not a major component in Danshen preparations. Therefore, the mechanism of inhibition of mOat1/mOat3-mediated transport of PAH/ES was investigated for LSA, RMA, and SAA (Table 1). Our previous work with CHO-mOat3 cells showed that ES accumulation was linear through the first 5 min and that ES exhibited a *K*_*m*_ of 12.2 ± 4.8 *μ*M [15]. In this study, time course evaluations in CHO-mOat1 cells indicated that PAH accumulation was linear through at least the first 5 min, and *K*_*m*_ was estimated as 13.0 ± 3.3 *μ*M (Figure 3). Using these parameter estimates as guidelines, kinetic experiment conditions were set to 1 *μ*M ES for 1 min and 5 *μ*M PAH for 2 min. The background corrected untransformed data for LSA, RMA, and SAA on mOat1 or mOat3 was fit to the mixed inhibition model, which incorporates competitive, noncompetitive, and uncompetitive inhibition modes. The estimated *α* values were found to be much larger than 1, indicating that inhibition of mOat1- and mOat3-mediated transport by LSA, RMA, and SAA was competitive in nature (Table 1).

### 3.3. Inhibition Potencies of LSA, RMA, and SAA

**Figure 4:**
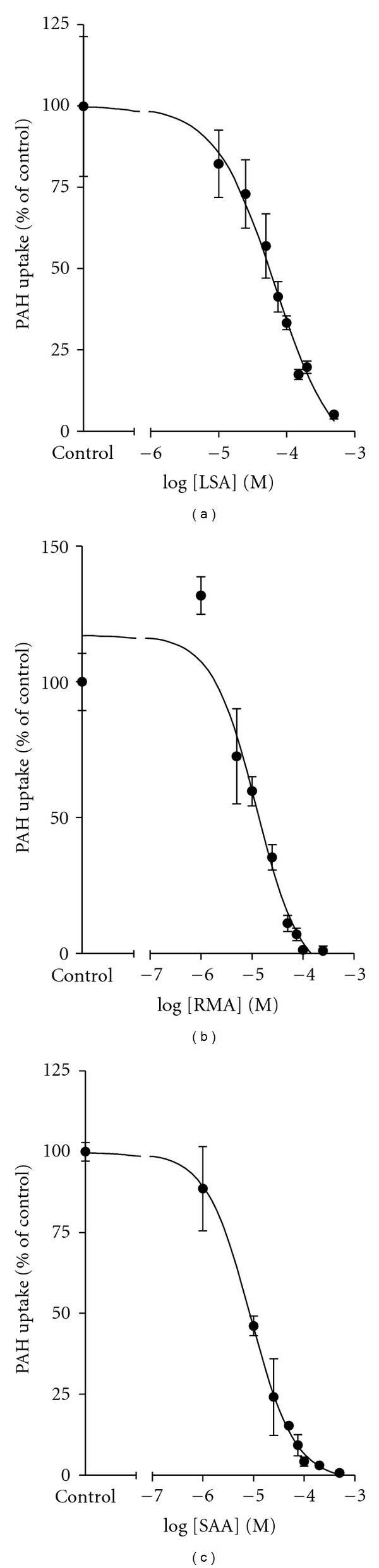
*K*_*i*_ determination for LSA, RMA, and SAA in the CHO-mOat1 cell line. Two minute uptake of [^3^H]PAH (5 *μ*M) in CHO-mOat1 cells was measured in the presence of increasing concentrations (10^−7^ to 5 × 10^−4 ^M) of LSA, RMA, and SAA. Data were corrected for nonspecific background measured in the CHO-FRT cells prior to kinetic analysis. *K*_*i*_ values were determined with non-linear regression and the “one-site competition” model using GraphPad Prism software. Experiments were repeated three times in triplicate with the mean *K*_*i*_ ± S.E.M. reported in Table 2. Graphs shown are from representative experiments with values plotted as mean ± S.D. (*n* = 3).

**Figure 5:**
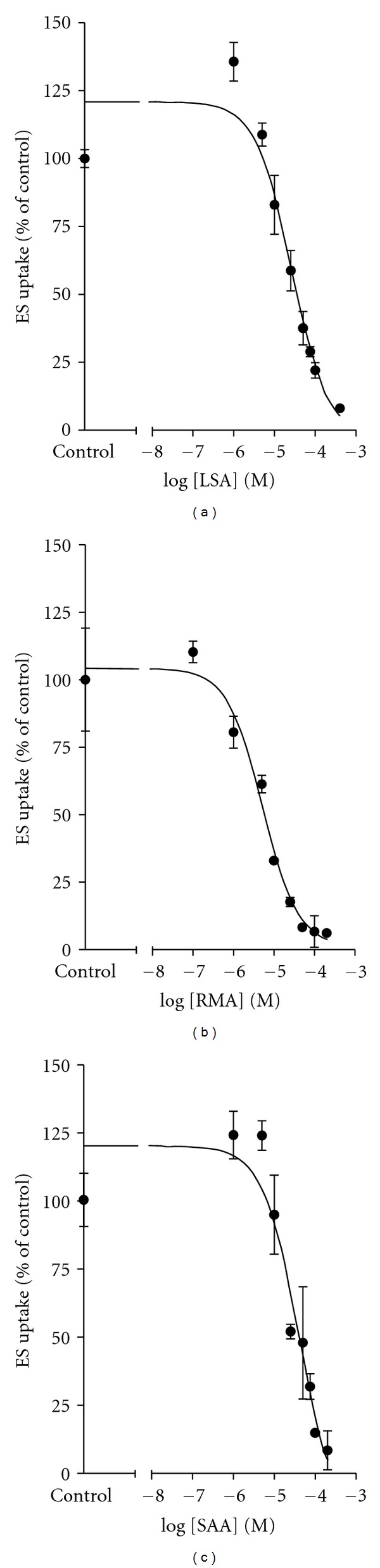
*K*_*i*_ determination for LSA, RMA, and SAA in the CHO-mOat3 cell line. One minute uptake of [^3^H]ES (1 *μ*M) in CHO-mOat3 cells was measured in the presence of increasing concentrations (10^−7^ to 4 × 10^−4 ^M) of LSA, RMA, and SAA. Data were corrected for nonspecific background measured in the CHO-FRT cells prior to kinetic analysis. *K*_*i*_ values were determined with non-linear regression and the “one-site competition” model using GraphPad Prism software. Experiments were repeated three times in triplicate with the mean *K*_*i*_ ± S.E.M. reported in Table 2. Graphs shown are from representative experiments with values plotted as mean ± S.D. (*n* = 3).

To allow direct comparison of inhibition potencies of LSA, RMA, and SAA for mOat1 and mOat3, experiments were conducted to determine the inhibition constant (*K*_*i*_) values (Figures 4 and 5 and Table 2). Since LSA, RMA, and SAA were found to be competitive inhibitors of mOat1- and mOat3-mediated transport, subsequent *K*_*i*_ analysis was performed using competitive inhibition. Applying increasing concentrations of unlabeled test compounds (10^−7^ to 5 × 10^−4 ^M), inhibition of mOat1- or mOat3-mediated transport was measured (Figures 4 and 5). Inhibition constants were estimated as 14.9 ± 4.9 *μ*M for LSA, 5.5 ± 2.2 *μ*M for RMA, and 4.9 ± 2.2 *μ*M for SAA on mOat1-mediated transport (Table 2). Values determined in the CHO-mOat3 cell line were 31.1 ± 7.0 *μ*M for LSA, 4.3 ± 0.2 *μ*M for RMA, and 21.3 ± 7.7 *μ*M for SAA (Table 2). For all of these analyses the coefficient of determination (*r* ^2^) was >0.9. No significant differences between *K*_*i*_ values for mOat1 and mOat3 were detected as determined by Student's unpaired *t*-test.

## 4. Discussion

Many natural products, which are extracted from living organisms, have beneficial biological or pharmacological activities and have been used as medicines throughout the world for more than 1,000 years. Today, they are often used as first-line therapeutics, dietary supplements, or complementary/alternative medicines, and it is reported that ~20% of adults in the United States are taking an herbal product [20]. Over the last decade a number of studies have identified transporter proteins, including OATs, as sites of drug-drug and natural product-drug interactions. For example, the dietary polyphenol, ellagic acid, which exhibits beneficial antioxidant and anticancer properties, was demonstrated to be one of the most potent inhibitors of hOAT1 (IC_50_ = 207 nM) identified to date [21]. Recently, the nephrotoxin aristolochic acid, which is produced by *Aristolochia *sp. plants, was identified as the causative agent for “Chinese herbs nephropathy” (now referred to as aristolochic acid nephropathy). When investigated, aristolochic acid was found to be a high-affinity substrate for both mOat1 (*K*_*m*_ = 790 nM) and mOat3 (*K*_*m*_ = 514 nM), suggesting that these transporters are key mediators in the renal proximal tubule cell accumulation of this toxicant [10, 22]. Thus, this interaction likely explains the biochemical mechanism underlying the acutely targeted, proximal tubule specific nature of aristolochic acid toxicity. Clearly, OATs expressed in the renal proximal tubule represent highly probable sites of natural product-drug (or natural product-endogenous substrate) interactions. Further studies such as these are needed in order to establish informed safety and efficacy profiles for herbal products.

Danshen, a traditional herbal medicine, continues to be used in the treatment of angina, myocardial ischemia, and other cardiovascular diseases throughout the world including Asia, Europe, and North America [1]. In 2010, the Danshen pharmaceutical product, Fufang Danshen Dripping Pill, successfully completed Phase II clinical trials in the United States (http://clinicaltrial.gov/ct2/show/NCT00797953?term=tasly&rank=1). To date, however, no formal studies examining the interaction between OATs and hydrophilic Danshen compounds, which are small organic acids and known to be major components in its pharmaceutical products, have been reported. Therefore, in the present study, we investigated whether any of the six purported active Danshen components, LSA, PCA, RMA, SAA, SAB, and TSL, interacted with the transporters mOat1 and/or mOat3 (Figures 1 and 2).

A major advantage of this study is that while Danshen preparations (and indeed the majority of herbal medicines) include multiple active components, purified preparations of individual Danshen extract components were used. Thus, the strength of interaction of each compound could be quantified independently versus the merged effects of a mixture containing all of the compounds. Similarly, since the transporters were expressed in isolation, the interaction of each individual compound with each individual transporter could be specifically assessed versus the *in vivo* situation where multiple transporter proteins are expressed simultaneously in a tissue, and the measured response is possibly an amalgamation of the activities of multiple transporter proteins. While under certain conditions all six compounds significantly inhibited mOat1- and mOat3-mediated transport, LSA, RMA, and SAA emerged as potent competitive inhibitors (Figures 4 and 5 and Tables 1 and 2). The rank order of their potencies was different between the two transporters with RMA *≈* SAA > LSA for mOat1 and RMA > SAA *≈* LSA for mOat3. While statistically not different, the *K*_*i*_ ratio (Table 2) suggests that mOat1 may have a slightly higher affinity (lower *K*_*i*_) for LSA and SAA than mOat3. However, in general, all of the values are quite similar and exhibit well below an order of magnitude in difference. The estimates are comparable to those reported for the strong OAT inhibitor, probenecid, *K*_*i*_ = 6.4 and 4.6 *μ*M for mOat1 and mOat3, respectively [7, 23, 24]. Thus, LSA, RMA, and SAA may affect the disposition and elimination of coadministered drugs that are also mOat1 and mOat3 substrates. This may be particularly relevant in patients suffering from coronary disease where Danshen pharmaceutical products could be combined with drugs such as angiotensin converting enzyme inhibitors, and studies with the human orthologs of these transporters need to be conducted.

*In vivo* pharmacokinetic studies have indicated that elimination of hydrophilic Danshen components involves active renal secretory pathways. In rats, the unbound renal clearance of TSL was ~5X greater than the glomerular filtration rate [25]. A separate study in rats found that spiking additional SAA into Danshen preparations increased the AUC of SAB (82.4%) and TSL (26.7%) and markedly reduced their clearances (46.8% and 32.9% for SAB and TSL, resp.) [26]. As we determined that SAA was a potent competitive mOat1/mOat3 inhibitor, and SAB and TSL can interact with mOat1 and mOat3, our data suggest that these *in vivo* results may be explained by competition for transport on Oat1/Oat3. An *in vivo* pilot study conducted in healthy humans reported *C*_max⁡_ values for LSA and RMA of 2.1 *μ*M and 6.7 *μ*M after *i.v. *dosing of a Danshen product [5]. These *C*_max⁡_ values are ~0.07–1.56-fold higher than the *K*_*i*_ values for mOat1 and mOat3 determined in the present study (Table 2). As recommended by the FDA Guidance for Drug Interaction Studies, when unbound *C*_max⁡_/IC_50_ (or *K*_*i*_) ≥ 0.1, it indicates that drug-drug interactions may occur *in vivo* [27]. Unfortunately, the plasma protein binding for LSA and RMA has not been reported, while TSL showed 25% plasma protein binding in rat plasma [25]. However, if we assume RMA to be highly protein bound (90%), the unbound *C*_max⁡_/*K*_*i*_ ratio for RMA would be 0.12 and 0.16 on mOat1 and mOat3, respectively, indicating potential for herb-drug interactions. Naturally, if human OAT orthologs show similar (or higher) affinities, possible herb-drug interactions should be considered.

A recent investigation indicated that flavonoid and hydroxycinnamic acid aglycones and their phase II metabolites (e.g., sulfate and glucuronide conjugates) significantly inhibit hOAT1 and hOAT3 transport activity [28, 29]. It is known that after oral administration in healthy volunteers, the majority of RMA exists as such conjugated forms, as well [30, 31]. Currently, there are no reports on characterization of LSA and SAA metabolites in human plasma, while SAA might undergo methylation and glucuronidation in rats [32]. Therefore, studies elucidating the metabolic pathways of these compounds are needed such that potential interactions of their metabolites with Oat1 and/or Oat3 can be examined.

In addition, there are other transporter families known to interact with small organic anions, for example, the multidrug resistance-associated proteins (MRPs, ABCC family) and the organic anion transporting polypeptides (OATPs, SLCO family). For the most part these latter two transporter families and the OATs have fairly distinct substrate interactions; however, the possibility that they also may contribute to the overall clinical pharmacokinetic profile of Danshen components* in vivo* needs to be considered until investigated directly. For example, it was recently suggested that SAB might be an OATP substrate based upon inhibition of total and biliary clearance of SAB by rifampicin, an OATP inhibitor, in rats [33].

Finally, the amount of each active component in Danshen preparations varies with different cultivation regions and manufacturing processes [4]. The variation of RMA in raw plant material was observed to be ~16-fold between different growing regions, and the dose of RMA ranged from 4.1 to 160 mg (~40 fold) in injectable dosage forms produced by different manufacturers [4, 5]. In a clinical study, the *C*_max⁡_ of RMA after *i.v. *infusion of a 160 mg dose was 1.2–1.6-fold higher than the *K*_*i*_ values for mOat1 and mOat3 [5]. These data suggest that, clinically, patients either would or would not have the potential for significant herb-drug interactions solely depending upon which manufacturer's product they were administered. Thus, although tanshinone II A, SAB, and protocatechuic aldehyde have been used as quality control markers in the manufacture of Danshen pharmaceutical dosage forms, the work presented here indicates that perhaps the content of LSA, RMA, and SAA should be monitored and controlled as well.

## Figures and Tables

**Table 1:** Estimated *α* values from mixed inhibition model analysis for LSA, RMA, and SAA.

Compound	mOat1	mOat3
LSA	6.7 × 10^11^	1.9 × 10^15^
RMA	1.3 × 10^12^	2.5 × 10^19^
SAA	2.6 × 10^14^	3.4 × 10^14^

Values are reported as mean. The S.E.M. is not applicable in this analysis.

**Table 2:** Estimated *K*_*i*_ (*μ*M) values for mOat1- and mOat3-mediated transport.

Compound	mOat1	mOat3	*K*_*i*_ ratio (mOat1/mOat3)
LSA	14.9 ± 4.9	31.1 ± 7.0	0.48
RMA	5.5 ± 2.2	4.3 ± 0.2	1.29
SAA	4.9 ± 2.2	21.3 ± 7.7	0.23

Values are reported as mean ± S.E.M. (*n* = 3). No significant differences between *K*_*i*_ values for mOat1 and mOat3 were detected (*P* < 0.05) as determined by Student's unpaired *t*-test.
